# Evolution of SARS-CoV-2 Variants: Implications on Immune Escape, Vaccination, Therapeutic and Diagnostic Strategies

**DOI:** 10.3390/v15040944

**Published:** 2023-04-10

**Authors:** Nur Zawanah Zabidi, Hern Liang Liew, Isra Ahmad Farouk, Ashwini Puniyamurti, Ashley Jia Wen Yip, Vindya Nilakshi Wijesinghe, Zheng Yao Low, Julian W. Tang, Vincent T. K. Chow, Sunil K. Lal

**Affiliations:** 1 School of Science, Monash University Malaysia, Subang Jaya 47500, Selangor, Malaysia; 2 Department of Respiratory Sciences, University of Leicester, Leicester LE1 7RH, UK; 3 Infectious Diseases Translational Research Program, Department of Microbiology and Immunology, Yong Loo Lin School of Medicine, National University of Singapore, Singapore 117545, Singapore; 4 Tropical Medicine & Biology Platform, Monash University, Subang Jaya 47500, Selangor, Malaysia

**Keywords:** coronaviruses, COVID-19, SARS-CoV-2, genetic variants, sub-variants, vaccine evasion, therapeutic approaches, diagnostic testing

## Abstract

The COVID-19 pandemic caused by SARS-CoV-2 is associated with a lower fatality rate than its SARS and MERS counterparts. However, the rapid evolution of SARS-CoV-2 has given rise to multiple variants with varying pathogenicity and transmissibility, such as the Delta and Omicron variants. Individuals with advanced age or underlying comorbidities, including hypertension, diabetes and cardiovascular diseases, are at a higher risk of increased disease severity. Hence, this has resulted in an urgent need for the development of better therapeutic and preventive approaches. This review describes the origin and evolution of human coronaviruses, particularly SARS-CoV-2 and its variants as well as sub-variants. Risk factors that contribute to disease severity and the implications of co-infections are also considered. In addition, various antiviral strategies against COVID-19, including novel and repurposed antiviral drugs targeting viral and host proteins, as well as immunotherapeutic strategies, are discussed. We critically evaluate strategies of current and emerging vaccines against SARS-CoV-2 and their efficacy, including immune evasion by new variants and sub-variants. The impact of SARS-CoV-2 evolution on COVID-19 diagnostic testing is also examined. Collectively, global research and public health authorities, along with all sectors of society, need to better prepare against upcoming variants and future coronavirus outbreaks.

## 1. Introduction

The global threat of the coronavirus disease 2019 (COVID-19) pandemic has accounted for about 680 million infected cases and 6.8 million deaths, as of March 2023 [1]. The etiological agent responsible for this catastrophic pandemic is SARS-CoV-2 (severe acute respiratory syndrome-coronavirus-2), the seventh known coronavirus to infect humans to date [2,3]. First reported in December 2019 in Hubei province, China, SARS-CoV-2 is a single-stranded, positive-sense RNA coronavirus with a genome of 29.9 kb that infects humans and animals, leading to respiratory illnesses ranging from mild symptoms to fatal outcomes [4,5]. SARS-CoV-2 belongs to the family *Coronaviridae*, which is divided into three subfamilies, *Letovirinae, Orthocoronavirinae* and *Pitovirinae*. The *Orthocoronavirinae* can be further classified into four different genera, namely the Alpha, Beta, Gamma and Delta coronaviruses [6,7]. Amongst these genera, certain betacoronaviruses are documented to be responsible for severe respiratory illness and death in humans [8].

The alphacoronavirus HCoV-229E and betacoronavirus HCoV-OC43 were the first known coronavirus species to infect humans [9,10]. Subsequently, other species were discovered over the years, including the alphacoronavirus HCoV-NL63 and betacoronaviruses HCoV-HKU1, SARS-CoV, MERS-CoV and SARS-CoV-2 [11]. Human coronaviruses NL63, 229E, OC43 and HKU1 can cause mild upper respiratory illnesses, common cold and pneumonia, especially in immunocompetent individuals [12,13,14]. HCoV-OC43 was first discovered in 1967, with its complete genomic profiling achieved in 2004 [15]. To date, there are eight genotypes (A-H), likely due to the natural genetic recombination over the years [16]. HCoV-229E was first discovered in 1965 and was postulated to be associated with bats [17,18,19]. While most HCoV-229E-infected patients manifest mild symptoms (e.g., common cold), younger, elderly and immunosuppressed individuals may suffer from life-threatening acute respiratory distress syndrome or ARDS [20,21]. With a seasonal cycle in temperate climates, HCoV-NL63 was first discovered in 2004 in a 7-month-old infant suffering from bronchiolitis and conjunctivitis [22]. Sources point toward bats as the potential reservoir [23]. HCoV-HKU1 was first discovered in 2004 in Hong Kong and thought to be associated with rodents rather than bats, compared with its betacoronavirus counterparts [24,25]. To date, there is incomplete information on morbidity and mortality rates, as well as geographical data of HCoV-NL63, HCoV-229E, HCoV-OC43 and HCoV-HKU1 infections. Nonetheless, they share the same mode of human-to-human transmission via airborne droplets expelled from the human respiratory tract or saliva, with some data on transmission via fomites [26,27,28]. SARS-CoV, MERS-CoV and SARS-CoV-2 betacoronaviruses are by far the major coronaviruses known to cause significant epidemics in humans.

SARS-CoV, MERS-CoV and SARS-CoV-2 are more pathogenic and infectious betacoronaviruses that may give rise to respiratory and other complications, and even death [29]. The SARS-CoV outbreak was reported in November 2002 in Guangdong province, China [30]. It progressed and spread rapidly to Hong Kong, Singapore, Vietnam and Canada by March 2003, with a fatality rate worldwide of about 10% [30,31]. The MERS-CoV outbreak was first reported in September 2012 in Jeddah, Saudi Arabia, and associated with an alarming fatality rate of 40%, with studies implicating camels as the reservoir [32,33]. SARS-CoV-2 was first reported in Wuhan, China, in December 2019. The primary reservoir is postulated to be bats, similar to SARS-CoV [34,35,36]. Despite causing a relatively lower fatality rate of 3.4%, the uncanny ability of SARS-CoV-2 to acquire mutations has resulted in the rapid emergence of numerous Variants of Concern (VOC) and Variants of Interest (VOI) with enhanced infective capacity due to factors such as stronger angiotensin-converting enzyme 2 (ACE2) receptor-binding affinity and heightened human-to-human transmissibility [37]. These variants have significantly diminished the efficacy of vaccines, thus escalating their threat to global public health [38]. For instance, vaccine protection against the B.1.351 (Beta) variant has been reduced, e.g., the efficacy of the Pfizer-BioNTech (BNT162b2) vaccine decreased from 95% to 75% in early 2021 [39].

SARS-CoV-2 is also transmitted via respiratory droplets and fomites. Common symptoms of COVID-19 include fever, dry cough, fatigue, dyspnea and headache [40,41,42]. Certain individuals may also suffer from common symptoms such as vomiting, diarrhea, skin lesions and conjunctivitis. Many patients have a diminished sense of smell and taste, namely anosmia and dysgeusia. A study on the symptom profile of infections with the SARS-CoV-2 variants showed that the prevalence of anosmia and dysgeusia symptoms differs by variant (i.e., 33.7% for the Delta variant, 13.4% for Omicron) [43,44]. Given that the ACE2 receptor is expressed in the sustentacular cells and glandular cells of the olfactory epithelium; inflammation and cytokine release (such as IL-6) upon infection alters neuronal signalling, leading to anosmia [45]. The ACE-2 receptor is also present in the tongue epithelium and can regulate taste perception, further alluding to the consequences of cytokine signalling on diminished taste upon SARS-CoV-2 infection [46]. The higher prevalence in Alpha, Beta, Gamma, and Delta variants may be attributed to the D614G mutation that promotes chemosensory epithelial cell infection—thereby impacting smell and taste damage [46]. The relatively lower anosmia prevalence of the Omicron variant may be due to differences in tissue tropism, i.e., its specific preference for cathepsin-mediated endosomal entry over the surface in TMPRSS2-mediated membrane fusion; thus sparing the highly expressed TMPRSS2 olfactory epithelium from infection [47]. Some patients suffer from long-term effects following COVID-19, which are ascribed by varying terminology, including long COVID; post-acute COVID-19 syndrome; and post-COVID-19 condition [48].

Risk factors of severe COVID-19 include unvaccinated individuals, advanced age [49], male gender [50,51] and underlying comorbidities such as hypertension, diabetes and cardiovascular diseases [52,53]. Additional environmental risk factors include crowding, poor ventilation [54,55] and certain occupations with high exposure to viruses and pathogens [55]. Moreover, co-infections with different SARS-CoV-2 variants and other pathogens have resulted in an urgent need for more effective and novel therapeutic approaches [56,57,58]. Since the beginning of this pandemic, new and/or repurposed vaccines and antiviral agents have been widely administered as preventive and therapeutic approaches [29]. However, with the emergence of new variants and the continuous increase in COVID-19 cases due to the diminished efficacy of vaccine-induced immune responses, the world is now faced with the challenge of confronting a moving target [39].

In efforts to curb the pandemic, it is imperative to unravel the genomic mutations of VOCs, highlight their changes in infectivity, and thus better understand the trajectory of SARS-CoV-2 evolution. This review also discusses the importance of different antiviral strategies comprising newly developed as well as repurposed antiviral drugs, including immunotherapeutic agents. In addition, the efficacies of vaccines against SARS-CoV-2 with respect to different viral variants, gender, age and adverse effects are also discussed. Seeking better insights into the above issues can provide solutions to help combat COVID-19. 

## 2. Variants and Sub-Variants of SARS-CoV-2

Changes in virus properties during the rapid evolution of SARS-CoV-2 may result in enhanced infection capacity accompanied by greater severity and death [59]. While not all SARS-CoV-2 variants share similar virulence, they inevitably affect the efficacy of antiviral agents and vaccines, thus rendering the management of the COVID-19 pandemic difficult [59]. To expedite research progress, the World Health Organization (WHO) has categorized the SARS-CoV-2 variants as VOC, VOI, or variants under monitoring (VUM), with the nomenclature of the former two based on the Greek alphabet (Table 1). As the name suggests, VOC dictates an alarming level of infection capacity, followed by a decrease in therapeutic efficacy globally. On the other hand, VOI denotes variants that cause significant community transmission in various regions that may lead to the next VOC [37]. VUM are variants that require enhanced monitoring pending new evidence for the classification of phenotypic or epidemiologic impact on the community.

As of December 2022, the Omicron variant represents the only remaining circulating VOC. The Alpha, Beta, Gamma and Delta variants are now classified as previously circulating VOCs [37]. The Alpha variant was first identified in the UK in December 2020 [60]. The significance of the Alpha variant is the N501Y mutation that enhances the binding affinity of the spike (S) protein to the human ACE2 receptor [61]. An additional P681H mutation provides an additional basic residue that may increase furin cleavage between the spike S1 and S2 subunits [62]. The E484K mutation found in sub-lineages of the Alpha variant is thought to interact with the K31 residue in the ACE2 receptor, further enhancing the binding affinity [63]. Moreover, E484K contributes to a significant loss of neutralizing activity of convalescent sera and monoclonal Ab (mAb) due to the disruption of the N-terminal domain (NTD) and receptor-binding domain (RBD) of the S protein. Thus, the BNT162b2 vaccine has up to four-fold reduced efficacy against the E484K B.1.1.7 variant [64].

Next, the Beta variant was first documented in South Africa in May 2020 [65]. Akin to the Alpha variant, the Beta variant carries the N501Y and E484K mutations. In addition, Beta harbors two additional significant mutations, i.e., K417N and L18F. The K417N mutation interacts with the ACE2 D30 residue, giving rise to enhanced binding affinity [66,67]. K417N also alters key interactions with class 1 neutralizing Ab (nAb), thus facilitating immune evasion [68,69]. The L18F mutation confers enhanced infection capacity with increased replication and Ab escape on NTD binding of the S protein [70,71]. 

The Gamma variant was first discovered amongst travellers from Brazil in January 2021 [72]. It possesses the K417N, E484K, N501Y and L18F mutations found in the Beta variant, with an additional P681H mutation found in the Alpha variant, rendering it more infectious due to enhanced ACE2 binding affinity to promote viral entry [61,62,72]. 

First discovered in India, the Delta variant was the major cause of numerous COVID-19 cases worldwide before the emergence of the Omicron variant [59]. The Delta variant carries T478K, P681R and L452R mutations in the S protein, with certain sub-variants (such as B.1.351 and P.1) possessing the K417N mutation [73]. The T478K mutation increases the electrostatic potential on the RBD of the S protein, facilitating immune evasion between the RBD and elicited Abs [74]. The P681R mutation enhances furin-mediated spike cleavage, thus promoting SARS-CoV-2 cell fusion [75]. The L452R mutation gives rise to reduced cell-mediated cellular immunity and enhanced binding affinity for ACE2 receptors by stabilizing the S protein, thus increasing viral replication and infection capacity [76]. The K417N mutation contributes to immune evasion and enhanced ACE2 binding affinity, giving rise to the Delta plus variant with a much higher infection capacity [77]. 

Following Delta, a novel variant with increased transmissibility was first detected in South Africa in November 2021. This variant B.1.1.529 was later designated as a VOC and named Omicron on 26 November 2021 [78]. To date, five major sub-lineages have been identified for the Omicron variant, i.e., BA.1, BA.2, BA.3, BA.4 and BA.5 [79,80,81]. The Omicron variant has overtaken Delta as the most widely circulated coronavirus variant. Initially, BA.1 and BA.2 were the most predominant sub-variants. The BA.4 and BA.5 subvariants were first identified in Botswana, Africa in early 2022, and subsequently superseded its earlier counterparts [82]. The Omicron variant has the highest number of mutations thus far, harboring about 50 mutations with over 30 mutations within the S protein [83]. Some of these include most of the key mutations in previous VOCs (Alpha, Beta, Gamma and Delta), such as K417N, E484A, D614G and N501Y, which elicit decreased nAb [84]. BA.1, BA.2 and BA.3 sub-variants possess approximately 39, 31 and 34 mutations in the S protein, respectively [80,81]. Amongst these three sub-variants, there are a total of 21 shared mutations, with BA.1 and BA.2 containing 13 and 8 unique alterations [85]. However, the genetic rearrangement of BA.2 is more pronounced due to the accumulation and loss of several mutations [86]. These mutations enable BA.2 to surpass BA.1 through immune escape and higher transmissibility. Examples of such unique mutations are V213G, R408S and T376A. Amongst the 21 common mutations of the Omicron sub-variants, a few notable examples include G339D, S477N and N440K [87,88]. The G339D mutation elicits a modest increase in binding affinity to the ACE2 receptor and is associated with the escape from a subset of nAb [87,89]. The S477N mutation increases viral infectivity via enhanced ACE2 receptor binding [68,88]. The N440K mutation confers additional resistance against certain mAb and is associated with the escape from several nAb, including those generated from vaccines [87]. 

On the other hand, VOIs possess genetic markers associated with diminished Ab sensitivity, and increased transmissibility or severity. They are identified as the cause of the increased prevalence of cases or outbreak clusters in certain regions, and have the potential to become future VOCs. There are eight previously circulating VOIs, i.e., the Epsilon, Zeta, Iota, Eta, Theta, Kappa, Lambda and Mu variants [37]. Amongst these, the Lambda and Mu variants represent the latest to be identified and are discussed below.

The Lambda variant was first identified in Peru in August 2020 [92]. This variant has seven mutations, of which three were novel, i.e., deletion Δ246-252, L452Q and F490S [95]. L452Q and F490S both map to the RBD of the S protein. L452Q is associated with increased ACE2 receptor binding affinity [91], whereas F490S is linked to reduced susceptibility towards Ab neutralization, particularly towards the Pfizer-BioNTech (BNT162b2) vaccine [92]. First detected in Columbia in January 2021, the Mu variant harbors a series of common mutations, such as E484K and N501Y in the RBD [90]. These mutations share the same characteristics as VOCs in terms of their increased affinity for the ACE2 receptor as well as reduced Ab sensitivity [96,97]. Other novel mutations in the Mu variant include the YY144-145TS and 146N insertion mutation in the NTD [97]. Insertion mutation 146N is reported to affect the S1 closed–open conformation, thus culminating in greater affinity for ACE2 receptor binding [98]. The remaining six VOIs each possess a set of defining mutations as well as common mutations that have been identified in other VOCs and VOIs.

The underlying mechanisms of SARS-CoV-2 evolution are of significant interest to better understand the factors that affect viral infectivity and virulence. Mutagenesis represents the primary source of viral evolution in which random mutations occur spontaneously in the genome due to limited fidelity errors or to adaptation to cellular environments imposed by host immune responses and selective pressures [99,100]. These mutations occasionally confer the ability to evade immune responses, enhance viral transmission and/or reduce vaccine efficacy [101,102]. Common mutation sites in SARS-CoV-2 include the RBD of the S protein, in which certain single nucleotide polymorphisms (SNPs) strengthen ACE2 receptor binding, thereby resulting in more infectious variants [103]. Such mutations occur to favor natural selection in which infectivity-strengthening mutations eventually outpace infectivity-weakening mutations [77,104]. A notable example of a pro-viral SNP is the D614G mutation. Variants harboring this mutation are more infectious due to enhanced viral entry, rendering them to be dominant global strains [104]. The mutation rate of SARS-CoV-2 is relatively low despite possessing a low-fidelity viral RNA polymerase that occasionally generates errors during transcription. Bioinformatics analyses of SARS-CoV-2 sequences indicate that the nucleotide mutation rate of the whole genome and of the S gene are about 6.7 × 10^−4^ and 8.1 × 10^−4^ substitution per site per year, respectively [105]. The generation of new variants worldwide is mainly contributed by the rapid spread of the virus within a vast population coupled with the prolonged duration of the pandemic [99,104,106]. Furthermore, co-infection of different strains may give rise to genetic recombinant strains retaining mutations and traits from both parental strains. A recombinant strain designated “XE” was detected in the UK in January 2022—it was found to be derived from the BA.1 and BA.2 Omicron sub-lineages [107]. XE contains BA.1 mutations in non-structural proteins (NSP) 1–6, as well as BA.2 mutations for the remaining genome [108]. Other recombinant strains include “XD” and “XF”, which are both products of recombination of the Omicron BA.1 and Delta variants [109]. To date, there are over 500 Omicron sub-variants. Many more are emerging worldwide due to mutagenesis and recombination to generate notable sub-variants, such as XBB BA.2.75 and BQ.1, which were widely reported in Singapore, India and USA [110]. Currently, the dominant Omicron sub-variant in many countries worldwide is XBB.1.5, which differs from XBB.1 by the presence of the F486P mutation in the spike protein. This mutation enhances ACE2 binding affinity and renders XBB.1.5 more infectious and transmissible but without change in disease severity [111]. Informally known as “Kraken”, XBB.1.5 also possesses highly antibody-evasive and immune escape properties [112]. Both the XBB and BQ.1 strains possess convergent mutations in hotspots such as R346T and F486S, which confer extreme immune-evasive ability against Omicron-specific Ab—thus, causing a surge in COVID-19 cases arising from breakthrough infections [93]. As for BA.2.75, the mutations G446S and R493Q significantly increase immune escape and ACE2 binding [94]. Other emerging variants with advantageous mutations are also of concern as they greatly impact the deployment and development of therapeutic strategies, including mAb therapy. 

## 3. Risk Factors for COVID-19 Severity

There is a need to revisit risk factors that contribute to disease manifestation and severity. Generally, the elderly and immunocompromised individuals are vulnerable to coronavirus infections [28]. Elderly persons are more prone to develop severe symptoms when infected [49]. For example, more than half of patients admitted into intensive care units (ICU) were ≥60 years old, which is associated with a higher mortality rate [50,113]. In contrast, younger individuals are less likely to be infected and tend to be asymptomatic or manifest mild symptoms when infected [114]. This difference may be explained by immunosenescence in the elderly—a weakened immune system exemplified by declines in natural killer cytotoxic activity, neutrophil activity, macrophage activation, T-cell production and pathogen recognition—which promotes viral replication [115]. Moreover, ageing is linked to a higher prevalence of comorbidities that lead to poorer outcomes. For instance, hypertension and cardiovascular diseases resulted in higher mortality rates [52,53].

Gender also exerts an important role in disease severity. Hence, males have a higher case fatality rate than females [50,51]. Moreover, the proportion of male COVID-19 patients in many studies comprised over half of the total cases, ranging from 51.4% to 73.2% [116]. The underlying reason may be attributed to the varying cellular immunity between males and females. Males are more susceptible to pathogens, whereas females tend to elicit a stronger antigenic response towards infections and vaccines [117]. Females also exhibit stronger T-cell responses with enhanced CD8+ T-cell activation [118]. Conversely, reduced T-cell activation and responses correlate with older age in males, leading to poorer disease outcomes [118]. 

As mentioned, individuals with comorbidities are more likely to experience severe symptoms. There is a higher prevalence of underlying medical conditions in COVID-19 patients in ICU compared to non-ICU patients [116]. One study noted that a higher number of patients infected with the Omicron variant is associated with comorbidities compared to the Delta variant [119]. Many critically ill COVID-19 patients are also diagnosed with comorbid conditions such as hypertension, cardiovascular disease, diabetic mellitus and metabolic syndrome. In these comorbidities, key mediators (including pro-inflammatory cytokines) are significantly dysregulated, resulting in altered Th1 and Th2 responses, impaired macrophage and lymphocyte functions and endothelial dysfunction—culminating in weakened immunity and greater disease severity. For instance, diabetic patients are less responsive to treatment, giving rise to a higher mortality rate due to abnormal lung function [120,121]. 

Furthermore, a correlation between COVID-19 severity and chronic obstructive pulmonary disease (COPD) was recently reviewed. COPD is characterized by lung damage that commonly arises from persistent exposure to noxious gases, such as cigarette smoke. Hence, COPD patients suffer from airway obstruction, exacerbations from respiratory tract infections and emphysema, all of which constitute risk factors for COVID-19 patients [122]. Additional conditions that COPD patients experience which may worsen the clinical outcomes of COVID-19 patients include: hypoxic pulmonary vasoconstriction, pulmonary thromboembolism and secondary bacterial infections [123]. Analysis of electronic patient records revealed high mortality and severe outcomes in COVID-19 patients with underlying COPD [124,125]. This association may be attributed to the elevated gene and protein expression of ACE2 in epithelial cells of COPD patients, accompanied by dampened host antiviral responses [126,127]. 

Other than COPD, obesity also constitutes a risk factor for COVID-19 patients. Obesity and extreme obesity correlate with COVID-19 hospitalizations and deaths, whereas being overweight will increase the risk of COVID-19 hospitalization only [128,129]. In one report, drastic increases in hospitalization and death rates, along with invasive mechanical ventilation and ICU admissions were observed in patients with higher body mass index (BMI)—about half of COVID-19 patients are obese [130]. COVID-19 patients with obesity experience a longer recovery time (20.6 versus 16.0 days), suggesting a higher viral load and slower antiviral response in obese patients [131]. Other contributory factors may include: increased resistance in the airway (obstructive sleep apnea), reduced lung volume and weaker respiratory muscles in obese patients [132]. Individuals with obesity also have higher levels of circulating pro-inflammatory cytokines (such as TNF-α, IL-6 and C-reactive protein), further aggravating the cytokine storm observed in COVID-19 patients [133]. Besides that, the activation of the renin-angiotensin-aldosterone system (RAAS) in obese patients can enhance ACE2 expression. Along with a higher amount of ACE2 expression in the adipose tissue, the above factors may enhance host susceptibility to COVID-19 [134].

Other than host risk factors, it is important to highlight environmental risk factors (including crowding, poor ventilation and hygiene, occupational hazards and frequent animal contact) that can potentially promote viral exposure and transmission [54,55]. The continuous emergence of SARS-CoV-2 variants with greater infection capacity and enhanced receptor binding pose a persistent threat to public health [135,136,137]. For instance, the CoronaVac/Sinovac inactivated virus vaccine showed only 50% protection against the P.1 variant [138]. The Pfizer-BioNTech mRNA and AstraZeneca ChAdOx1 vaccines also exhibit up to an 11-fold reduction in sensitivity against the B.1.1.7 variant, and only about 10% protection against the B.1.351 variant [139,140]. Primary immunization with two doses of AstraZeneca or Pfizer-BioNTech vaccine confers little protection against the Omicron variant. Although the third dose conferred slightly increased efficacy, its effect waned over time [141]. The vaccine efficacy of primary immunization prevented hospital admissions by 85% during the Alpha and Delta VOC period, and by 65% during the Omicron VOC period [142]. One study [143] revealed that vaccinated Omicron-infected individuals are less likely to require ICU admissions compared to vaccinated Delta-infected individuals (0.47% versus 1.00%). However, hospitalized Omicron-infected patients may experience symptoms as severe as Delta-infected patients [119,143]. Although some protection is conferred to vaccinees, non-vaccinated individuals remain at higher risk of infection, particularly towards variants with enhanced infection capacity.

## 4. Occurrence and Impact of Co-Infections and Super-Infections

Co-infections occur when more than one infectious agent is simultaneously present in the host. However, the risk of increased or decreased severity of co-infections is controversial. Co-infection of viruses can lead to the common outcome of viral interference, i.e., suppressed replication of one virus by another virus competitively [144]. Different pathogens (viruses, bacteria, fungi, parasites) may also co-infect, and may alter the disease epidemiology, morbidity and mortality [145]. Co-infections of viral strains may increase the risk of genetic recombination, resulting in new variants. This may impact drug and vaccine efficacy, potentially causing the emergence of resistant strains [146]. Thus, there is a need to identify and study these interactions in greater detail, as they can pose a long-term threat to public health.

As for SARS-CoV-2 co-infections, there are limited data, and the risk of disease severity remains inconclusive. A systematic review and meta-analysis [56] correlated co-infection and super-infection with their outcomes in COVID-19 patients. In this study, co-infection was defined as the “recovery of other respiratory pathogens in COVID-19 patients at the time of diagnosis”, whereas super-infection was the “subsequent recovery of other respiratory pathogens during the care of SARS-CoV-2 infection”. About one fifth of patients were co-infected with other pathogens, with a higher prevalence amongst non-ICU patients. On the other hand, a prevalence rate of 24% was reported for super-infections, with 41% being ICU patients. The common bacterial co-pathogens in these patients were *Klebsiella pneumoniae*, *Streptococcus pneumoniae*, *Staphylococcus aureus* and *Acinetobacter*. Influenza A, influenza B and respiratory syncytial virus (RSV) were the most common co-infecting viruses in patients with SARS-CoV-2 infection [56]. In another meta-analysis [57], hospitalized COVID-19 patients across multiple countries showed incidence rates of 7% and 3% for bacterial and viral co-infections, respectively. This difference in incidence may be attributed to the control of respiratory viruses that share similar transmission routes through the implementation of social distancing and mask-wearing [147]. Bacterial or fungal co-infections are potentially more harmful in COVID-19 patients in the ICU compared to respiratory virus co-infections [148]. COVID-19 patients co-infected with tuberculosis (TB) also exhibit weaker immune responses against SARS-CoV-2 [149]. Co-infections of dengue and SARS-CoV-2 viruses also pose a threat in dengue-endemic countries and may lead to high mortality [150,151]. 

Patients with co-infections are admitted to ICU more frequently, while those with super-infections have longer hospital stay and increased mortality rate [152]. While there are reports of viral co-infection, most studies indicate that bacterial co-infection is more frequent in hospitalized patients with viral respiratory tract infection [153]. 

Interestingly, individuals can be co-infected with multiple strains of SARS-CoV-2 [58]. A study of 19 SARS-CoV-2-positive samples revealed that all samples had co-infection with more than one strain classified as type-A and type-B strains [154]. Three strains from type-A (S1A, S3A, S6A) can be traced back to the G clade, while the other strains belong to unique clades outside of the five commonly known SARS-CoV-2 clades. Another study found that 5% of the population had co-infection with two or more SARS-CoV-2 variants from different clades [155]. Thus, there was a different pattern of co-infection with samples of the same clades. For example, two clusters were observed under clade 19B, i.e., one cluster comprised multiple infections from various clades (19A, 19B, 20A, 20B), while the other cluster consisted of clades 19B and 20A. This reiterates that SARS-CoV-2 has a large genome diversity, and co-infection of multiple strains may lead to genetic recombination and the eventual emergence of novel strains [156]. Hence, the newly detected “Delmicron” variant is thought to be a recombinant of the Omicron and Delta variants [157]. Therefore, it is important to be cognizant of such potential recombination events and the possible emergence of “super variant(s)”. 

More conclusive studies on the implications and effects of co-infections and super-infections on disease severity are warranted in order to improve clinical guidelines and measures and to achieve better outcomes. 

## 5. Antiviral Strategies against COVID-19

Medical interventions against COVID-19 include therapeutic treatments such as antiviral drugs and immunotherapy, as well as prophylactic interventions such as vaccination. 

### 5.1. Antiviral Drugs

There are two direct-acting antiviral drugs that have been developed for the treatment of COVID-19. Molnupiravir (Lagevrio^®^) is an isopropyl ester prodrug which targets the viral RNA-dependent RNA polymerase (RdRp). Its antiviral activity is mediated via a mutagenesis mechanism that misincorporates adenine or guanine triphosphates, ultimately yielding grossly mutated and dysfunctional viral RNA [158,159,160,161]. Treatment with molnupiravir reduces hospitalization or death risks in mild to moderate COVID-19. It maintains efficacy regardless of the timing of symptom onset, underlying risk factors and variant type (Alpha, Delta, Gamma, Mu, Omicron) [162,163,164,165]. For patients at risk of high disease severity, early molnupiravir treatments within 3 days from symptom onset diminishes disease progression [166]. However, certain issues remain to be addressed and resolved, including adverse effects, correlation with vaccination, antiviral resistance, cost-effectiveness and accessibility. For example, one study suggests that molnupiravir treatment has induced the evolution of viral strains harboring multiple mutations that may be capable of transmission to other individuals. In view of these potential risks of this mutagenic agent to generate novel viral variants that may be immune-evasive, there are concerns and calls to halt its clinical use [167].

Formerly used for the treatment of SARS-CoV infection, nirmatrelvir (Paxlovid^®^) is an inhibitor of Mpro (3CLpro), a major SARS-CoV-2 protease that mediates the proteolytic cleavage of polyproteins pp1a and pp1ab into NSPs that are essential for viral replication [168]. Nirmatrelvir binds covalently to the Mpro catalytic cysteine within the active site [169]. This antiviral can significantly reduce the risk of hospitalization or death in patients with severe COVID-19 [170]. A combination therapy with ritonavir further decreases the metabolism of nirmatrelvir to increase the overall exposure and reduce the drug dosage. Early treatment with nirmatrelvir/ritonavir within 5 days of symptom onset has been reported to prevent disease progression in high-risk patients with no need for oxygen supplementation [171]. However, in vitro passaging experiments of SARS-CoV-2 in nirmatrelvir reveal that SARS-CoV-2 resistance to nirmatrelvir may readily arise via multiple pathways, e.g., conferred via E166V mutation [172]. The highly conserved Mpro binding-pocket residues have also motivated research into alternative antiviral drugs that share the same target, e.g., masitinib [169,173,174]. Ensitrelvir is also an inhibitor of SARS-CoV-2 3CLpro—clinical trial data reveal that this novel antiviral can shorten the symptoms of mild to moderate COVID-19 and significantly reduce the number of days that patients test positive for the virus [175].

### 5.2. Immunotherapy

Immunotherapy is an effective therapeutic strategy to combat viral diseases, as shown through reduced mortality rates in SARS and MERS [176,177]. mAbs are a form of passive immunotherapy designed to specifically bind to the target epitope region of a specific host or viral protein. The mAbs can mimic, block or induce other changes to trigger mechanisms that intervene with the viral life-cycle [178]. According to regulations stipulated by WHO, mAb development includes validation by in vitro and in vivo studies, as well as clinical trials to determine its pharmacodynamics, pharmacokinetics and safety profile [179]. Due to the critical need for therapeutic treatments during the surging COVID-19 pandemic, several mAbs such as LY-CoV555 (bamlavinimab), REGN10933 (casirivimab) and REGN10987 (imdevimab) were formerly approved by the US Food, Drug and Administration (FDA) under emergency use authorization [180,181]. However, based on updated data reflecting unlikely efficacy against the Omicron variant, authorization of their prescription has been revised. For example, the co-administration of bamlanivimab and etesevimab or casirivimab and imdevimab is now limited to patients who are exposed to or infected with variants susceptible to these treatments [182]. In vitro, in vivo and clinical studies on mAbs developed to treat SARS-CoV-2 infection are summarized in Table 2. Amongst these, some have proceeded to clinical trials approved by the US National Institutes of Health (Table 3).

The mAbs may lose their efficacy via viral mutations that cause conformational changes in target epitopes. The mechanism underlying SARS-CoV-2 neutralization by newly designed mAbs entails disrupting the interaction between the S protein and ACE2 receptor for viral entry. The target region of these mAbs predominantly spans specific amino acid sequences of the S protein, mainly within the RBD or receptor-binding motif (RBM). Binding to RBD indirectly inhibits the S protein–host receptor interaction by inducing a pre-to-post-fusion conformational change in the S protein. This mode of action is demonstrated by mAbs REGN10987, 47D11, B38 and H4 [193,194,195]. Binding to the RBM, as shown by mAbs REGN10933 and 2B04, directly blocks the virus–host interaction [196,197]. Although RBM-targeting mAbs have higher potency, their specificity may lead to poor binding and limited cross-neutralization due to the low sequence conservation of the S protein [198]. 

As reflected in Table 2, different mAbs exhibit varying neutralizing efficacies across the SARS-CoV-2 variants. The mAbs used as monotherapy suffer the risk of loss in potency against circulating resistant variants due to pre-existing mutants in the quasispecies at low frequency [199]. Furthermore, there may be a rapid selection of escape mutations de novo, such as E484K, E484Q and L452R, which gave rise to the Beta, Kappa (B.1.617.1) and Delta variants, respectively [200]. Hence, the clinical use of LY-CoV555 as monotherapy has been revoked due to its inability to reduce infection by several VOCs such as the Beta variant [181,197]. There are more reports on the evasion of mAb-induced protection against novel SARS-CoV-2 variants and sub-variants, although the magnitude of neutralization reduction varies among mAb–variant pairs. For example, sotrovimab retained its in vitro neutralization capacity against Omicron BA.1 but exhibited diminished efficacy against BA.2, BA.4 and BA.5 sub-variants [201]. Furthermore, the Omicron BQ.1.1 and XBB sub-lineages possess greater immune-evasive capabilities than earlier Omicron variants such as BA.2 and BA.5—this highlights the continuous need for new therapeutic mAbs and strategies against COVID-19 [202].

Alternatively, cocktail mAb therapeutic strategies utilizing two or more mAbs may compensate for any loss in neutralization potency. Nonetheless, the viral resistance profile of each component must be considered. For example, a clinical study comparing the LY-CoV555/LY-CoV016 and REGN10933/REGN10987 combination therapies revealed the former having a higher risk of hospitalization and death than the latter, possibly due to resistance of viral strains containing E484 and K417 mutations [190]. However, the latter combination displayed efficacy in both vaccinated and unvaccinated individuals [188]. Another example is cilgavimab/tixagevimab (Evusheld^TM^), the only option available for pre-exposure prophylaxis (PrEP) of COVID-19 for immunocompromised individuals who are unable to attain adequate immune response upon vaccination [203]. With updated data demonstrating its unlikely efficacy against variants responsible for 90% of infections in the USA, the FDA has restricted its use based on the proportion of non-susceptible SARS-CoV-2 variants nationally is ≤90% [204]. Despite substantial financial costs and labor investment in developing mAbs, they play important roles, particularly in patients admitted to the ICU or individuals unable to elicit immune responses upon vaccination, such as the elderly and immunocompromised. 

Another option of passive immunotherapy is convalescent plasma (CP) therapy, in which nAbs are transferred from the blood of an individual who has recovered from COVID-19. Some studies on CP therapy in critically ill COVID-19 patients show clinical improvement without any serious adverse effects [205,206,207]. However, many studies were inappropriately designed, terminated early or did not examine the effects of co-treatments, baseline levels of pre-existing nAbs, disease severity and timing of administration. 

Ultimately, extreme caution is advised against the unrestricted use of CP therapy since there remain gaps in research studies. It is crucial that future clinical trials are better designed and randomized, recruit larger patient cohorts with differing disease stages and evaluate their applicability in healthcare systems of low- to high-income countries [208].

### 5.3. Drug Repurposing

#### 5.3.1. Viral Proteins as Antiviral Targets

Remdesivir is an example of an antiviral drug that has been actively repurposed against COVID-19. This drug retained antiviral activity against Alpha, Beta, Gamma, Delta and Omicron VOCs in Vero E6-GFP cells [165]. In clinical studies involving infected patients without the need for supplemental oxygen, but who are at high risk of disease progression, a 3-day course of remdesivir is sufficient to achieve lower hospitalization and death rates [209,210,211]. Remdesivir is also the only antiviral agent approved for hospitalized patients on non-invasive oxygen support. When administered for 5 days, patients achieved higher recovery and hospital discharge rates, along with a reduced risk of serious adverse events as well as the requirement for supplemental oxygen and invasive ventilation [212,213]. However, there is potential for the resistance of remdesivir since decreasing sensitivity was observed for NSP12 E802D mutations in vitro [214]. This has been validated in an immunocompromised patient with persistent SARS-CoV-2 infection, strongly implying that remdesivir can exert selective pressure in vivo to drive viral evolution [215]. 

To address this issue, clinical trials have evaluated the combination therapy of other agents. Lopinavir/ritonavir (Kaletra^®^) acts as a protease inhibitor by dysregulating the viral structural and functional proteins to inhibit viral replication. This combination was first reported to lower the body temperature and restore normal physiological function after COVID-19 infection [216]. Other clinical studies found neither significant decreases in viral load [217] nor benefits beyond the standard of care in hospitalized patients of high disease severity [218]. Darunavir, another protease inhibitor structurally similar to lopinavir, has also been tested in combination with cobicistat against COVID-19. Compared to lopinavir/ritonavir, it was instead found to increase the risk of death [219]. Interferon-β1b (Extavia^®^) acts as an immune system booster by stimulating innate antiviral responses. Interferon-β1b can promote recovery and reduce the risk of intubation or death in patients with severe COVID-19 [220]. However, its highly variable response rate and potential side-effects may restrict its usage. Compared with placebo, early treatment with pegylated interferon lambda can reduce viral loads by day 7 in COVID-19 patients with high baseline viral load, and significantly decrease hospitalization [221].

#### 5.3.2. Host Proteins as Drug Targets

To overcome antiviral resistance, there are alternative therapeutic approaches targeting host proteins instead of viral proteins to modulate the host immune system [222]. Their efficacy is attributed to the more conserved nature of host proteins. Some therapeutic candidates include corticosteroids, mAbs, antidepressants and anticoagulants. Dexamethasone is a synthetic corticosteroid with broad-spectrum immunosuppressant functions. It inhibits cytokine secretion and is therefore useful in counteracting COVID-19-related hyperinflammation and cytokine storm. Dexamethasone reduces mortality, particularly in hospitalized patients on supplementary oxygen therapy or with inflammatory lung injury [223]. Its selective usage is recommended only in intubated patients with severe disease since dexamethasone may interfere with the protective function of T-cells, Ab production and macrophage clearance—which may lead to higher plasma viral load and susceptibility to secondary infection [223,224]. 

Tocilizumab, IC14, leronlimab and eculizumab are repurposed mAbs that target immune pathways linked to COVID-19. Tocilizumab targets the IL-6 receptor and can achieve clinical improvement and ameliorate disease progression. When administered intravenously, treatment outcomes in COVID-19 patients with pneumonia were mixed, as elevated IL-6 levels are only detectable in critical and severe infections [225]. When administered subcutaneously, rapid improvements in clinical outcomes and inflammation biomarkers were attained, suggesting its use at a lower dosage for patients with less disease severity [226]. IC14 binds and inhibits CD14, which facilitates the blocking of multiple inflammatory responses during COVID-19 infection [227,228]. IC14 thus diminishes neutrophil and protein concentrations that contribute to acute lung injury [188]. Leronlimab binds to the CCR5 receptor to increase CD8 T-cells, while decreasing inflammatory cytokines and viral RNA [229]. Eculizumab exerts indirect immunoprotective and immunoregulatory effects by binding to complement protein C5 to inhibit its cleavage to C5a and C5b. This prevents the generation of terminal complement complex C5b-9, which mediates cellular lysis [230]. After four infusions of eculizumab, infected patients with severe pneumonia or ARDS experienced successful recovery with a decrease in inflammatory biomarkers such as C-reactive protein [231].

The antidepressant fluvoxamine is a strong S1R agonist that increased survival and reduced likelihood of clinical deterioration in clinical trials of hospitalized COVID-19 patients [232,233]. Its pharmacological actions may be supported by its ability to stimulate the host σ-1 receptor, which regulates cytokine secretion [232]. The anticoagulant heparin has been found to destabilize the interaction between RBD of the S protein and ACE2, resulting in decreased respiratory distress, as well as reduced risks of hospitalization and mortality [234,235,236]. However, heparin resistance has been observed in patients with critical infections thus, necessitating further investigations into an ideal management plan for thromboprophylaxis [237].

## 6. Impact of SARS-CoV-2 Evolution on Efficacy of Vaccinations

Recognition of the significance of the S protein included the discovery of its role in membrane fusion and binding to ACE2 [238,239,240]. In addition, the role of nAb and specific recognition of the RBD of S protein provided great success in rendering the S protein as a significant target for vaccine design [241,242]. In response to the COVID-19 pandemic, there has been remarkable and unprecedented progress in the development and deployment of robust vaccine candidates against SARS-CoV-2. Current vaccination strategies include mRNA-based vaccines, inactivated whole virus vaccines, vector vaccines (e.g., adenovirus vector vaccines), subunit vaccines, virus-like particles and live attenuated vaccines.

To minimize the spread of COVID-19 and achieve herd immunity, adolescents and children must also be immunized since they contribute significantly to virus transmission. Initially, the FDA approved the emergency use of the Pfizer vaccine for individuals under the age of 18 [243]. The Pfizer vaccine was then approved for emergency use in children over 6 months [244]—e.g., a clinical vaccination trial showed 90.7% efficacy in preventing COVID-19 in 3100 children, with only mild to moderate side-effects. Moderna vaccine trials involving children and adolescents yielded similar results [245], and the Moderna vaccine is currently also available to those over the age of 6 months [243].

Unvaccinated individuals incur a higher risk of testing positive and dying from COVID-19 compared to fully vaccinated persons. Continuous immunization programs will limit SARS-CoV-2 transmission and result in fewer hospitalizations and deaths. Vaccinations continue to be an important and critical strategy in efforts to end the COVID-19 pandemic. To date, over 13 billion COVID-19 vaccine doses have been administered worldwide. Overall, reports of significant adverse events post-vaccination are relatively rare. Given that the current vaccines are authorized for emergency use, their effects necessitate close monitoring to better understand their long-term safety profiles. According to the US Centers for Disease Control and Prevention (CDC), the vaccines are effective, although fully vaccinated individuals may still be vulnerable to infection with newer VOCs [246]. With the emergence of SARS-CoV-2 variants, it appears that the efficacy of vaccines has been preserved against the Alpha variant, but their efficacy is reduced against the Beta, Delta and Omicron variants. Nevertheless, mass vaccination has decreased disease severity and hospitalizations. Booster doses of mRNA vaccines can restore their effectiveness against infection with the Omicron variant and risk of hospitalization, although this efficacy wanes over time. The Beta and Omicron variants exhibit immune escape abilities, but Omicron has become more prevalent due to its high transmissibility. Despite the various VOCs, studies claim that T-cell responses are sustained across all vaccines [247]. However, should future variants diminish vaccine efficacy, existing formulations may necessitate modification and adoption of newer approaches. The WHO expert group has proposed the development of monovalent and polyvalent vaccines that target single and multiple variants, respectively. In response, vaccine producers have initiated such efforts in compliance with regulatory criteria [247,248]. Pfizer-BioNTech is developing variant-specific vaccines (Alpha, Beta, Delta) as well as multivalent vaccines (bivalent Alpha/Delta and Alpha/Omicron), in which booster doses of all but the Beta-specific vaccine have shown significantly higher neutralizing activity against the Omicron variant compared to the original vaccine [249]. Animal studies show that booster doses of an Omicron-specific vaccine offer more or less the same level of protection compared to booster doses of the original Pfizer and Moderna vaccines [250]. Animal studies show that booster doses of bivalent COVID-19 vaccines that contain mRNAs encoding Omicron BA.1 or BA.4/5 SARS-CoV-2 spike proteins can enhance immunogenicity and protective efficacy against the BA.5 variant in mice [251]. Bivalent booster vaccines confer additional protection against symptomatic infections caused by Omicron sub-lineages related to BA.5, XBB and XBB.1.5 in persons who had previously received 2, 3 or 4 monovalent vaccine doses. Hence, the advisory that everyone who is eligible should stay up-to-date with recommended COVID-19 vaccines [252]. Adapted versions of the Oxford–AstraZeneca vaccine targeting the Beta and Omicron variants are also being formulated, as are Omicron-specific versions of the Sputnik V and Novavax vaccines [250]. It is imperative to continue to gather as much data as possible on the real-world efficacies of these variant-specific vaccines to evaluate the ongoing vaccine and booster implementation programs [253].

To complement the existing vaccines, the ongoing development of novel vaccines is crucial in the battle against COVID-19. However, their efficacy is challenged by constant and dynamic viral evolution. Despite administering vaccines to the general population, the unrelenting mutation of viral strains can confer the ability to evade vaccine-induced Abs. These viral mutants cause breakthrough infections in a fraction of vaccine recipients—it is thus concerning that vaccinated individuals may remain susceptible to SARS-CoV-2 infection. The emergence of vaccine-evasive viral strains would inevitably lead to new outbreaks of infection. In view of its evolutionary rate, it is possible for SARS-CoV-2 to further acquire mutations that render current vaccines and therapeutic Abs ineffective. An example of this phenomenon is the Omicron variant with its highly mutated RBD of the S protein [254]. The continuous evolution of SARS-CoV-2 variants (especially Omicron) can culminate in convergent mutations which evade neutralizing antibodies and convalescent plasma, while still maintaining ACE2 binding affinity. Due to humoral immune imprinting, Omicron BA.5 and BA.2 breakthrough infections decrease the diversity of neutralizing antibody-binding sites and increase the proportion of non-neutralizing antibodies—this concentrates humoral immune pressure and promotes convergent evolution in the RBD. Mutations on the RBD converge on several hotspots—as few as six additional mutations on BA.5 (i.e., R346, K356, K444, L452, N460K, F486) can completely evade antibodies in most plasma samples tested [93].

There is a proposal by the FDA to update COVID-19 vaccines annually, similar to the approach to updating influenza vaccines. However, there are differences in opinion in the scientific community on specific issues such as the optimal composition, frequency and timing of vaccination [255]. Universal or pan SARS-CoV-2 vaccines are currently undergoing preclinical and/or clinical testing and would represent an ideal long-term option to confer protection against future variants [248,256]. Therefore, a deeper understanding of the evolutionary trends of the virus is essential to devise more effective strategies to better prepare against the potential emergence of more infectious and lethal strains. 

## 7. Impact of SARS-CoV-2 Evolution on COVID-19 Diagnostic Testing

The rapid and ongoing evolution of SARS-CoV-2 precludes the recommendation of any particular diagnostic test which may have already been outdated by the time this text is published. Therefore, only principles of diagnostic testing will be discussed here. 

In general, diagnostic testing in any laboratory is limited by the facilities, manpower and budget available, but also the aims of the testing. In a hospital or clinic setting, testing for SARS-CoV-2 tends to fall into one of the following categories: to confirm a clinical suspicion of COVID-19 for patient triage, isolation and possible treatment (using antigen or PCR testing); for infection control purposes (using antigen or PCR and sometimes serology tests)—including discharge testing (using antigen or PCR); or to check for any vaccine or naturally-induced immunity (using serology). Testing during an outbreak includes these categories as well. Several national health institutions (e.g., in USA, UK, Germany) have attempted to update and maintain lists of effective test kits for various SARS-CoV-2 variants as they emerge [257,258,259]. However, for individual laboratories, the above practical limitations will govern the types of diagnostic tests they can actually implement.

SARS-CoV-2 PCR and lateral flow antigen testing (LFAT) are often used to screen symptomatic patients and their contacts—some of whom may be pre-infected or asymptomatically infected. Neither test can detect the virus while it is incubating and replicating intracellularly during the “eclipse” phase of the viral life-cycle. Many studies have shown that self-swabbing by such patients is effective and sensitive [260,261,262,263]. Advice and studies on the use of commercial PCR kits require users to check the target of the test against the prevalent circulating strains of the virus (currently Omicron) to assess its sensitivity and specificity [258]. For example, the TaqPath assay (ThermoFisher Scientific, Oxford, UK) fails to detect the S gene 69–70 deletion (the so-called “S gene target failure” or SGTF found in the Alpha and Omicron BA.1 and BA.3 variants) [264], but not in the Delta variant which does not contain this mutation. This allows the TaqPath assay SGTF feature to quickly identify and distinguish between these specific variants by process of exclusion [265]. However, other assays have since been developed to overcome this issue to specifically detect both Omicron BA.1 and BA.2 variants more directly [266].

It is important for local diagnostic laboratories to be aware of which SARS-CoV-2 variants are circulating locally within their population. This will facilitate in selecting an appropriate diagnostic kit and platform that can be further optimized for detection. In response to the continuously evolving virus, constant monitoring is vital to ensure that the most optimal test is used. Selecting a PCR test that targets non-S gene sites (e.g., E, N, other ORF gene targets) can reduce the risk of new viral S gene variant mutations that lead to “misses” on the assay, rendering false negative results [258]. Even though PCR assays are very sensitive to single nucleotide changes in their primer and/or probe binding sites, LFATs are more robust as they rely on a larger scale of antigen protein detection, typically the SARS-CoV-2 N protein. Here, minor nucleotide changes may not matter as much. Despite some variation and reduced sensitivity compared to test performances against the earlier Delta variant, most LFATs in current use are capable of detecting most Omicron variants [267,268,269]. Additional PCR testing can further validate a negative LFAT test in a patient with COVID-19-compatible symptoms. To avoid “misses” caused by mutations in the same SARS-CoV-2 gene region, it is suggested that PCR and LFAT tests use different targets.

Serological testing is also performed in healthcare settings to check for previous vaccination or natural infection, often as a (relatively variable) surrogate marker of immunity. Most serology kits target the viral N or S protein Ab responses [270]. As most of the current first generation of COVID-19 vaccines induce immunity only against the viral S protein, anti-N IgG assays will indicate whether or not a natural infection has occurred. An exception to this is if the population has been vaccinated using a whole virus vaccine (e.g., CoronaVac, Sinovac) which will also induce anti-N IgG Abs [271]. Serological responses take 5–10 days to evolve in SARS-CoV-2-naive individuals and are thus not used to test acutely symptomatic cases. Instead, they are more useful in population sero-surveys to assess the overall exposure to SARS-CoV-2, providing a crude estimate of population immunity, particularly those who have been relatively asymptomatic and may not have presented for PCR and/or LFAT testing [272,273].

As SARS-CoV-2 continues to evolve throughout the global population, many commercial kit manufacturers have included SARS-CoV-2 in their existing respiratory PCR test panels, along with other seasonal respiratory viruses (e.g., influenza, RSV, parainfluenza, adenoviruses) [274]. Furthermore, the level of containment for routine SARS-CoV-2 diagnostic testing has been gradually reduced from BSL-3 to BSL-2 [275,276] due to increased vaccination amongst healthcare workers, widespread availability of more effective antivirals, and the evolution of the virus into a milder phenotype in vaccinated populations.

## 8. Conclusions

In view of the rapid emergence of SARS-CoV-2 variants, it is important to understand the significance of viral evolution that leads to mutations with different infection capacities, virulence and disease severity. New mutations, such as those in the most recent Omicron variant and sub-variants, are a natural phenomenon in which the virus undergoes adaptation to host environments or arise from random replication errors. Considering that mutations can evade the immune system and diminish the efficacy of existing vaccines, it is imperative to unravel the underlying mechanisms to better detect and manage these emerging variants. Co-infections and interactions of different pathogens can also contribute to poorer disease outcomes, increased disease severity and mortality rates. Indeed, there are reports of co-infection of SARS-CoV-2 with other bacterial and viral pathogens, or even two or more SARS-CoV-2 variants. 

There are currently a number of drugs used to treat COVID-19, such as molnupiravir, nirmatrelvir and immunotherapeutic mAbs that target the S protein. However, there are major concerns and issues, such as antiviral resistance across variants, adverse effects, timing of therapy and disease severity. Newer therapeutic strategies, such as host-targeted therapeutics that obviate the risk of antiviral resistance, are also being exploited. Such efforts generate novel drugs with broad-spectrum capabilities to better manage viral infections. In response to the COVID-19 pandemic, there has been substantial progress in the development of robust vaccine candidates against SARS-CoV-2. Vaccines are now available for individuals aged 6 months and above. Overall, these vaccines are generally safe and effective in preventing the spread of COVID-19, and vaccination should thus remain a priority. To further identify potential vaccine candidates, future research can adopt in silico approaches through reverse vaccinology. Continuous global efforts, including surveillance, must be sustained to understand in depth the complex evolution of SARS-CoV-2 and to design better preventive strategies to exit this pandemic.

## Figures and Tables

**Table 1:** Significance of key mutations found in SARS-CoV-2 VOIs and VOCs.

Key Mutations	Significance	VOC or VOI	Reference
N501Y	Increases ACE2 receptor binding affinity	Alpha *, Beta *, Gamma *, Mu **, Omicron	[61,66,72,84,90]
P681H	Affects furin cleavage site between S1 and S2 subunits in the S protein, promoting viral entry into cells	Alpha *, Gamma *, Omicron	[62,72,83]
E484K	Contributes to considerable loss of neutralizing activity of convalescent sera and mAb Increases ACE2 receptor binding affinity through interaction with K31 residue	Alpha *, Beta *, Gamma *, Mu **	[63,66,72,90]
K417N	Increases ACE2 receptor binding affinity through interaction with D30 residue Alters the key interactions associated with class 1 nAb, contributing to immune evasion	Beta *, Gamma *, Delta *, Omicron	[66,72,77,87]
L18F	Associated with the escape in numerous NTD binding mAb and reduces neutralization by several antibodies (Abs)	Beta *, Gamma *	[66,72]
T478K	Increases electrostatic potential and interferes with the spike-RBD interaction with convalescent sera or vaccine-elicited Abs, contributing to immune evasion	Delta *, Omicron	[74,81]
P681R	Enhances furin-mediated spike cleavage, accelerating SARS-CoV-2 fusion to cell	Delta *	[75]
L452R	Increases ACE2 receptor binding affinity Promotes stability of the S protein Reduces cell-mediated immunity, causing rapid viral replication	Delta *	[76]
L542Q	Immune escape Increases ACE2 receptor binding affinity	Lambda **	[91]
F490S	Reduces susceptibility to Ab neutralization	Lambda **	[92]
D614G	Increases ACE2 receptor binding affinity Reduces S1 shedding and increases total S protein incorporated into the virion, enhancing virus infection	Alpha *, Beta *, Gamma *, Delta *, Omicron, Lambda **	[66,72,73,84,88,91]
R346K	Reduces binding of Abs with the RBD of SARS-CoV-2	Mu **, Omicron	[90]
ins146N	Affects the closed–open conformation of S1 subunit, leading to greater ACE2 receptor binding affinity	Mu **	[90]
G339D	Slightly increases ACE2 receptor binding affinity Associated with escape from a subset of nAb	Omicron	[87]
S477N	Increases viral infectivity through enhanced ACE2 receptor binding	Omicron	[88]
N440K	Confers high resistance towards certain mAb Associated with escape from several nAb, including those generated from vaccines	Omicron	[87]
R346T	Reduces neutralizing efficacy of vaccine-generated Abs	Omicron	[93]
F486S	Increases neutralization resistance of Abs Enhances fusogenicity	Omicron	[93]
G446S	Contributes to resistance against Abs generated from existing vaccines	Omicron	[94]
R493Q	Enhances binding to ACE2 receptor, eases adherence to cells	Omicron	[94]

Note: * previously circulating VOC; ** previously circulating VOI.

**Table 2:** Summary of results of treatments with monoclonal antibodies under in vitro, in vivo and clinical settings.

Year	Strategy	Stage	Study	Route of Administration	Virus Subtype	Dose	Outcome	Limitations	Ref.
2022	Cilgavimab/ Tixagevimab	Clinical	63 adult kidney transplant recipients	Intra-muscular	B.1.1.529 (Omicron)	300 mg	Low levels of neutralizing activity 29 days post-injection (n = 6; 9% of sample size)	High inter-individual variability due to patient’s body mass index	[183]
		In Vitro	Vero E6 cells		B.1.617.2 (Delta)	IC_50_ 10^1^ μg/mL	Partial neutralizing activity against Omicron (233-fold less active than against Delta)	Limited efficiency for immunocompromised patients	[184]
					B.1.1.529 (Omicron)				
2022	Sotrovimab	Clinical (Meta-analysis)	27,429 Infected patients	Intravenous	B.1.617.2 (Delta)	500 mg	Significantly reduced need for mechanical ventilation, ICU admission, hospitalization rate and mortality rate	No significant difference in disease progression, emergency department visit and incidence of adverse events	[185]
					B.1.1.529 (Omicron)				
		Clinical	3 infected unvaccinated patients, 3 infected partially vaccinated patients, 2 infected completely vaccinated patients	Intravenous	B.1.617.2 (Delta)	500 mg	Increased resistance mutations within the spike gene at positions S: E340K/A/V and P337L/T/S	Small sample size: Patient cohort initially involved 100 individuals, in which 23 had persistent infection post-treatment, 68 did not have a follow-up detection test, and only 8 had respiratory specimens available pre- and post-treatment	[186]
		In Vitro	Vero E6 cells		B.1.1.529 (Omicron)	IC_50_ 10^2^ ng/mL	Three-fold reduced potency against Omicron and Omicron-R346K variant VSV pseudoviruses compared to other mAbs (i.e., VR-7832, CT-P59, REGN10933, REGN10987, LY-CoV555, LY-CoV016) which completely lost their neutralizing activity Less than two-fold reduction in neutralizing activity against authentic Omicron SARS-CoV-2 compared to Wuhan-Hu-1		[187]
2021	REGN10933 + REGN10987 (Casirivimab/ Imdevimab) combination therapy	Clinical	630 Infected vaccinated and unvaccinated patients	NS	B.1.617.2 (Delta)	NS	Reduced hospitalization rates amongst vaccinated and unvaccinated patients	Cohort of patients tested during a Delta surge—variant not routinely sequenced for verification	[188]
		In Vivo	K18-hACE2 mice	Intra-peritoneal	Wash SA-B.1.351	2 mg/kg	Lower ability to reduce viral RNA levels in nasal washes Reduced levels of pro-inflammatory cytokines and chemokines		[189]
					Wash BR-B.1.1.28				
					WA1 2020 N501Y/D614G		Reduced levels of pro-inflammatory cytokines and chemokines		
					B.1.1.7 (Alpha)				
2021	REGN10933 (Casirivimab)	In Vitro	Vero-TMPRSS2 cells		Wash SA-B.1.351	EC_50_ 9462 ng/mL	Marked loss or complete absence of neutralizing activity		[189]
					Wash BR-B.1.1.28	EC_50_ 5002 ng/mL			
					D614G mutation	EC_50_ 7 ng/mL			
					N501Y/D614G mutation	EC_50_ 12 ng/mL			
					B.1.1.7 (Alpha)	EC_50_ 8 ng/mL			
					B.1.429 (Epsilon)	EC_50_ 4 ng/mL			
					B.1.617.1	EC_50_ 133 ng/mL			
					B.1.526 (S477N mutation)	EC_50_ 4 ng/mL			
					B.1.526 (E848K mutation)	EC_50_ 82 ng/mL			
2021	Comparative study of LY-CoV555 + LY-CoV016 (Bamlanivimab/ Etesevimab) and REGN10933 + REGN10987 (Casirivimab/ Imdevimab) combination therapy	Clinical	105 Infected patients	Intravenous	B.1.1.7 (Alpha)	LY-COV555 700 mg; LY-CoV016 1400 mg; REGN10933 1200 mg; REGN10987 1200 mg	No differences in primary endpoint	Small sample size Observational (non-randomized study) Selection bias—Choice of administration made based on drug availability	[190]
			43 Infected patients		P.1 (Gamma)		Bamlanivimab/etesevimab: Higher risk of hospitalization/death Casirivimab/imdevimab: Acted as a protective factor, reduced risk of disease progression		
2021	LY-CoV555 (Bamlanivimab)	In Vivo	K18-hACE2 mice	Intra-peritoneal	Wash SA-B.1.351	2 mg/kg	No virological protection in lungs, nasal washes and brain		[189]
					Wash BR-B.1.1.28				
					WA1 2020 N501Y/D614G		Reduced levels of pro-inflammatory cytokines and chemokines		
					B.1.1.7 (Alpha)				
2021		In Vitro	Vero-TMPRSS2 cells		Wash SA-B.1.351	EC_50_ > 10,000 ng/mL	Marked loss or complete absence of neutralizing activity		[189]
					Wash BR-B.1.1.28	EC_50_ > 10,000 ng/mL			
					D614G mutation	EC_50_ 5 ng/mL			
					N501Y/D614G mutation	EC_50_ 8 ng/mL			
					B.1.1.7 (Alpha)	EC_50_ 4 ng/mL			
					B.1.429 (Epsilon)	EC_50_ > 10,000 ng/mL			
					B.1.617.1	EC_50_ > 10,000 ng/mL			
					B.1.526 (S477N mutation)	EC_50_ 2 ng/mL			
					B.1.526 (E848K mutation)	EC_50_ > 10,000 ng/mL			
2021	2B04/47D11 combination therapy	In Vivo	K18-hACE2 mice	Intra-peritoneal	Wash SA-B.1.351	2 mg/kg	Better reduction of viral RNA in lungs than in nasal washes and brain		[189]
					Wash BR-B.1.1.28		Impaired reduction of viral burden in lungs, nasal washes and brain		
					WA1 2020 N501Y/D614G		Better reduction of viral RNA in lungs than in nasal washes Reduced levels of pro-inflammatory cytokines and chemokines		
					B.1.1.7 (Alpha)		Reduced levels of pro-inflammatory cytokines and chemokines		
2021		In Vitro	Vero-TMPRSS2 cells		Wash SA-B.1.351	EC_50_ 431 ng/mL	Poorer neutralization activity due to 47D11 mAb component		[189]
					Wash BR-B.1.1.28	EC_50_ 384 ng/mL			
					D614G mutation	EC_50_ 3 ng/mL	Efficient neutralization		
					N501Y/D614G mutation	EC_50_ 3 ng/mL			
					B.1.1.7 (Alpha)	EC_50_ 2 ng/mL			
					B.1.429 (Epsilon)	EC_50_ 4 ng/mL			
					B.1.617.1	EC_50_ 2187 ng/mL			
					B.1.526 (S477N mutation)	EC_50_ 1 ng/mL			
					B.1.526 (E848K mutation)	EC_50_ 644 ng/mL			
2021	2B04	In Vitro	Vero-TMPRSS2 cells		Wash SA-B.1.351	EC_50_ > 10,000 ng/mL	Marked loss or complete absence of neutralizing activity		[189]
					Wash BR-B.1.1.28	EC_50_ > 10,000 ng/mL			
					D614G mutation	EC_50_ 1 ng/mL			
					N501Y/D614G mutation	EC_50_ 1 ng/mL			
					B.1.1.7 (Alpha)	EC_50_ 1 ng/mL			
					B.1.429 (Epsilon)	EC_50_ 3 ng/mL			
					B.1.617.1	EC_50_ > 10,000 ng/mL			
					B.1.526 (S477N mutation)	EC_50_ 0.1 ng/mL			
					B.1.526 (E848K mutation)	EC_50_ > 10,000 ng/mL			
2021	47D11	In Vitro	Vero-TMPRSS2 cells		Wash SA-B.1.351	EC_50_ 240 ng/mL	Few changes in potency		[189]
					Wash BR-B.1.1.28	EC_50_ 277 ng/mL			
					D614G mutation	EC_50_ 319 ng/mL			
					N501Y/D614G mutation	EC_50_ 657 ng/mL			
					B.1.1.7 (Alpha)	EC_50_ 305 ng/mL			
					B.1.429 (Epsilon)	EC_50_ 456 ng/mL			
					B.1.617.1	EC_50_ 1091 ng/mL			
					B.1.526 (S477N mutation)	EC_50_ 130 ng/mL			
					B.1.526 (E848K mutation)	EC_50_ 341 ng/mL			
2021	DZIF-10c	In Vivo	NRG and huFcRn mice	Intranasal, intra-peritoneal	BavPat1/2020	40 mg/kg	Induced high neutralizing Ab titers Prolonged stability Favorable pharmacokinetic profile	Route of delivery may have had substantial impact on bioavailability and clinical efficacy	[191]
		In Vivo	hACE2-transduced BALB/c mice				Reduced histopathology and viral load reduction		
					B.1 (BavPat1)	IC_100_ 0.01 μg/mL			
					B.1.1.7 (Alpha)	IC_100_ 0.014 μg/mL	Remained fully active		
					B.1.351 (Beta)	IC_100_ 0.17 μg/mL	Retained activity, but with reduced potency		
2020	B38, H4	In Vivo	hACE2 mice	Intra-peritoneal	BetaCoV/ Shenzhen/ SZTH-003/2020	25 mg/kg	Conferred protective efficacy No lesions observed under administration of B38	Mild bronchopneumonia under administration of H4	[192]
		In Vitro	Vero E6 cells			B38 IC_50_ 0.177 μg/mL; H4 IC_50_ 0.896 μg/mL	Exhibited neutralizing activity in Vero E6 cells		
		In Vitro	Vero E6 cells		SARS-CoV-2/01/ human/Jan2020/ Thailand	B38 IC_50_ 5.45 μg/mL; H4 IC_50_ 0.492 μg/mL	H4 had better binding to RBD of S protein and neutralizing activity than B38		[193]

Note: NS = Not specified.

**Table 3:** Clinical trials on monoclonal antibodies as therapeutic strategy against COVID-19.

Clinical Trial Identifier	Sponsor	Phase	Status	Duration	Number of Participants	Route of Administration	Patient Criteria	Objective	Measured Outcomes
NCT05074433	Regeneron Pharmaceuticals (USA)	III	Completed	October 2021–May 2022	66	Subcutaneous	Uninfected child/adult (≥12 years old) Immunocompromised	Evaluate safety and efficacy of REGN10933 + REGN10987 (casirivimab/imdevimab) combination therapy as a pre-exposure prophylactic treatment	Symptom development Changes in anti-drug Ab/nAb to each mAb Incidence of adverse events
NCT04518410	National Institute of Allergy and Infectious Diseases (USA)	II/III	Active	August 2020–June 2023	4044	IV infusion	Infected adult (≥18 years old)	Evaluate safety and efficacy of REGN10933 + REGN10987 (casirivimab/imdevimab) combination therapy administered via intravenous infusion in infected adults who do not currently need hospitalization	Duration/severity of symptoms Viral load Oxygen saturation level Time to recovery Risk of hospitalization Incidence of adverse events Death rate
NCT04631705	University of Cologne (Germany)	I/II	Completed	December 2020–September 2021	45	Inhalation	Infected adult (18–70 years old)	Assess safety, pharmacokinetics, immunogenicity, and antiviral activity of DZIF-10c through inhaled administration	Pharmacokinetics Changes in anti-drug Ab Viral load Incidence of adverse events
NCT04644120	AbbVie (USA)	I	Completed	December 2020–August 2021	25	IV infusion	Infected adult (≥18 years old)	Evaluate safety, tolerability, and pharmacokinetics of ABBV-47D11 and ABBV-2B04 when given alone or in combination via intravenous infusion	Pharmacokinetics (maximum serum concentration, half-life) Changes in anti-drug Ab/nAb to each mAb Viral load Incidence of adverse events

## Data Availability

Not applicable.
